# EpiExplorer: live exploration and global analysis of large epigenomic datasets

**DOI:** 10.1186/gb-2012-13-10-r96

**Published:** 2012-10-03

**Authors:** Konstantin Halachev, Hannah Bast, Felipe Albrecht, Thomas Lengauer, Christoph Bock

**Affiliations:** 1 Max Planck Institute for Informatics, Campus E1.4, 66123 Saarbrücken, Germany; 2 Department of Computer Science, University of Freiburg, Georges-Köhler-Allee 51, 79110 Freiburg, Germany; 3 CeMM Research Center for Molecular Medicine of the Austrian Academy of Sciences, Lazarettgasse 14, 1090 Vienna, Austria; 4 Department of Laboratory Medicine, Medical University of Vienna, Währinger Gürtel 18-20, 1090 Vienna, Austria

## Abstract

Epigenome mapping consortia are generating resources of tremendous value for studying epigenetic regulation. To maximize their utility and impact, new tools are needed that facilitate interactive analysis of epigenome datasets. Here we describe EpiExplorer, a web tool for exploring genome and epigenome data on a genomic scale. We demonstrate EpiExplorer's utility by describing a hypothesis-generating analysis of DNA hydroxymethylation in relation to public reference maps of the human epigenome. All EpiExplorer analyses are performed dynamically within seconds, using an efficient and versatile text indexing scheme that we introduce to bioinformatics. EpiExplorer is available at http://epiexplorer.mpi-inf.mpg.de.

## Rationale

Understanding gene regulation is an important goal in biomedical research. Historically, much of what we know about regulatory mechanisms has been discovered by mechanism-focused studies on a small set of model genes [1,2]. High-throughput genomic mapping technologies have recently emerged as a complementary approach [3]; and large-scale community projects are now generating comprehensive maps of genetic and epigenetic regulation for the human and mouse genomes [4-7]. Substantial potential for discovery lies in better connecting mechanism-focused studies to the wealth of functional genomics and epigenomics data that are being generated. A handful of pilot studies highlight the value of combining high-throughput and mechanism-focused research (for example, in [8-10]), but few research groups are equally proficient in bioinformatics, large-scale genomics and in-depth functional analysis to conduct highly integrated studies of gene regulation. A new generation of software tools could bridge this gap by enabling user-friendly navigation and analysis of large genomic databases.

Genome browsers are currently the only software tools for navigating through genome data that are widely used, not only by bioinformaticians but also by biomedical researchers with little computational background. The strength of web tools such as the UCSC Genome Browser [11], Ensembl [12] and the WashU Human Epigenome Browser [13] lies in their intuitive interface, which allows users to browse through the genome by representing it as a one-dimensional map with various annotation tracks. This approach is powerful for visualizing individual gene loci, but the key concept of genomics - investigating many genomic regions in concert - tends to get lost when working with genome browsers only. Therefore, complementary tools are needed that handle the complexity of large genomic datasets while maintaining the interactive and user-friendly character of genome browsers.

Existing tools do not fully address this need. For example, the UCSC Table Browser [14] and Ensembl BioMarts [15] provide user-friendly support for selecting and downloading sets of genomic regions, but the analysis of the downloaded data needs to be performed locally using command-line tools, including BEDTools [16] and R/Bioconductor [17]. Workflow tools such as Galaxy [18], Taverna [19] and the Genomic HyperBrowser [20] combine user-friendliness and flexibility, but they require careful planning and tend to be too slow for performing truly interactive and exploratory analyses. Finally, enrichment analysis servers such as GREAT [21] and EpiGRAPH [22] are powerful tools for identifying significant associations in large biological datasets, but they lack the flexibility to explore the observed enrichments in a dynamic and interactive fashion.

With EpiExplorer, we have developed a web server that combines the interactive nature of genome browsers with the region-based analytical approach of Galaxy, enabling users to casually explore large-scale genomic datasets in search of interesting functional associations. EpiExplorer does not aim to replace any existing tool; instead it facilitates dynamic integration with tools such as the UCSC Genome Browser, Galaxy and the Genomic HyperBrowser. Neither does EpiExplorer restrict the user as to how to search for relevant associations in the data - as enrichment analysis tools do with their stringent statistical framework. Instead, EpiExplorer's key strength lies in supporting exploratory hypothesis generation using a broad range of genomic analyses performed in real time over the Internet. Such exploratory analyses often provide a first indication of relevant associations that are worth following up by in-depth statistical analysis using other software tools or by experimental validation in the wet lab.

## Software and applications

### A method and software for genome-wide exploration and live analysis of large epigenomic datasets

The EpiExplorer web server provides an interactive gateway for exploring large-scale reference maps of the human and mouse genome. EpiExplorer is built around default and user-uploaded genomic region sets, which are supplied as BED files. Before uploading data for EpiExplorer analysis, it is often useful to preprocess raw data with application-specific tools. For example, ChIP-seq data may be preprocessed with Cistrome [23] in order to derive a list of high-confidence peaks for the transcription factor or epigenetic mark of interest. Similarly, RNA-seq data may be preprocessed using Galaxy [18] in order to identify genomic regions that are differentially transcribed between two cell types.

Once the most meaningful BED file representation of the dataset of interest has been obtained, this list of genomic regions can be uploaded into EpiExplorer and interactively explored for hypothesis generation and visual analysis. The uploaded genomic regions are internally annotated with a wide range of genomic attributes, which enables visualization, analysis and filtering in real time. Five types of genomic regions are available in EpiExplorer by default, namely CpG islands, gene promoters, transcription start sites, predicted enhancer elements and a map of 5-kb tiling regions spanning the entire genome. Furthermore, EpiExplorer's default genomic attribute database includes chromatin and transcription factor binding data from the ENCODE project [6], epigenome data from the Roadmap Epigenomics Initiative [5], gene annotations from Gene Ontology [24] and Online Mendelian Inheritance in Man (OMIM) [25], and genome annotations from the UCSC Genome Browser [11]. Importantly, EpiExplorer makes it easy for users to upload their own sets of genomic regions and to use them with the same flexibility as any of EpiExplorer's default region sets.

We validated the utility of EpiExplorer by studying the genome and epigenome characteristics of CpG islands, which is a well-understood topic [26]. As outlined in a case study (see Text S1 and Figure S1 in Additional file 1) and its corresponding online tutorial on the supplementary website [27], EpiExplorer makes it easy to rediscover the distinctive epigenetic characteristics of CpG islands, which have previously been studied using computational and experimental methods [28-31]. The entire analysis can be performed in less than ten minutes without any bioinformatic training, guided by EpiExplorer's context-specific visualizations.

### Connecting a new epigenetic mark to large-scale reference maps of the human epigenome

To assess the utility of EpiExplorer for exploratory analysis and hypothesis generation in a more advanced setting, we investigated a recently discovered epigenetic mark. 5-Hydroxymethylcytosine (5hmC) is a chemical variant of normal (that is, non-hydroxylated) cytosine methylation. It was first observed in embryonic stem (ES) cells and in certain types of neurons [32,33]. The conversion of cytosine methylation into 5hmC is catalyzed by proteins of the TET family. One TET protein (TET2) is frequently mutated in myeloid cancers [34], underlining the biomedical relevance of studying the role of 5hmC in gene regulation.

**Figure 1:**
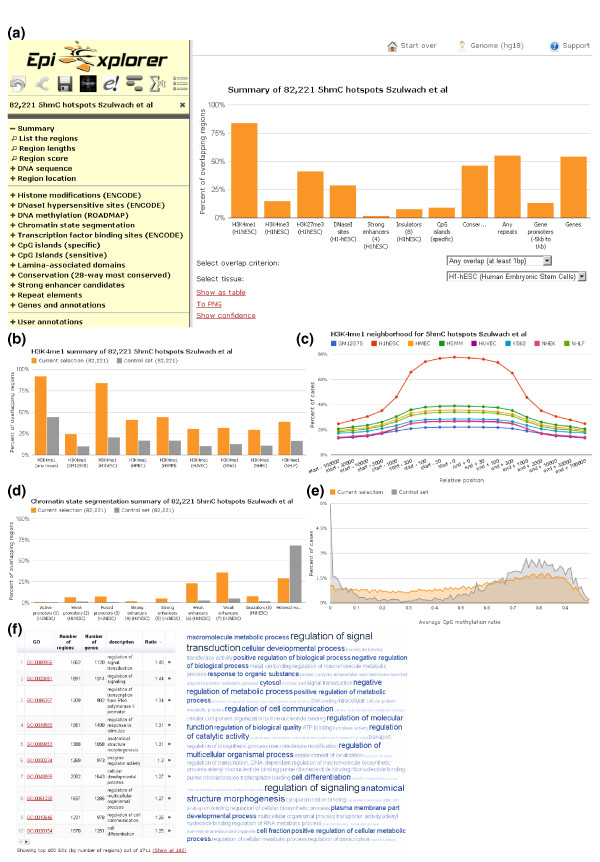
**Utilizing EpiExplorer for interactive analysis and hypothesis generation**. After uploading a set of published 5-hydroxymethylcytosine (5hmC) hotspots [35] into EpiExplorer, various options for genome-wide analysis are available. All diagrams are generated dynamically in response to user interactions. **(a) **Bar chart summarizing the percent overlap (y-axis) between 5hmC hotspots and various genomic datasets (x-axis) in H1hESC cells. **(b) **Bar chart comparing the percent overlap of 5hmC hotspots (orange) and randomized control regions (grey) with histone H3K4me1 peaks, based on ENCODE data [60]. **(c) **Genomic neighborhood plot illustrating the percent overlap (y-axis) with H3K4me1 peaks in the vicinity of 5hmC hotspots (x-axis). Different line colors correspond to H3K4me1 data for different cell types. **(d) **Bar chart comparing the percent overlap of 5hmC hotspots (orange) and randomized control regions (grey) with a comprehensive catalog of epigenetic states derived by computational segmentation of ENCODE histone modification data [39]. **(e) **Histogram illustrating the distribution of DNA methylation levels among 5hmC hotspots (orange) and randomized control regions (grey), based on Roadmap Epigenomics data [52]. **(f) **Enrichment table (left) and word cloud (right) illustrating the most highly enriched Gene Ontology (GO) terms among genes whose transcribed region is within 10 kb of a 5hmC hotspot. The most general (more than 5,000 associated genes) and most specific GO terms (less than 50 associated genes) were suppressed in this analysis.

From the paper of Szulwach *et al. *[35], we obtained the genomic region coordinates for a total of 82,221 hotspots of 5hmC that the authors experimentally mapped in human ES cells. We uploaded these hotspot regions into EpiExplorer, where they are automatically annotated with default genomic attributes such as gene annotations and associated epigenetic marks. EpiExplorer's initial overview screen summarizes the overlap of 5hmC hotspots with the most relevant genomic attributes and provides the starting point for interactive exploration of the dataset (Figure 1a). This view is tissue-specific, and we select a human ES cell line ('H1hESC') as the tissue type of interest. In ES cells, we observe striking overlap between 5hmC hotspots and epigenetic marks associated with distal gene-regulatory activity. Specifically, more than 80% of 5hmC hotspots overlap with peaks of the histone H3K4me1 mark, which is a well-known signature of enhancer elements [36]. In contrast, less than 20% of 5hmC hotspots overlap with histone H3K4me3 (Figure 1a), which is considered the hallmark of active core promoter regions [37].

To assess whether the association of 5hmC hotspots with H3K4me1 peaks indeed constitutes a relevant enrichment, we performed the same comparison for a randomized control set. EpiExplorer automatically calculates such control sets for user-uploaded region sets, which is done by reshuffling the genomic positions while retaining the overall number of regions and the distribution of region sizes. Visual comparison shows that the overlap between 5hmC hotspots and H3K4me1 peaks is indeed fourfold higher than expected by chance (Figure 1b), constituting a strong enrichment with potential biological implications. This enrichment is much more pronounced for H3K4me1 in ES cells than for other tissues, supporting the specificity of the observed association. We could further validate this association using EpiExplorer's neighborhood plot, which uses a similar concept as in the WashU Human Epigenome Browser [13] and in EpiChIP [38] in order to visualize the shared epigenomic neighborhood among a set of regions (Figure 1c). When plotting the levels of H3K4me1 methylation in the vicinity of 5hmC hotspots across the genome, we again observed a much stronger enrichment for ES cells than for H3K4me1 data from other tissues. Furthermore, when we compared the 5hmC hotspots with a comprehensive catalog of epigenetic states [39], we detected striking enrichment for several classes of enhancer elements (Figure 1d). In summary, these results suggest the hypothesis that a specific association may exist between 5hmC and H3K4me1-marked enhancer elements in human ES cells.

Given the presumed role of 5hmC in the erasure of DNA methylation [40,41], we also investigated the distribution of normal (that is, non-hydroxylated) cytosine methylation among 5hmC hotspots, again in comparison with the randomized control set. To that end, we use the ability of EpiExplorer to work on dynamically refined subsets of the data and filter the set of 5hmC hotspots down to those regions for which we also have sufficient DNA methylation data. The results show that 5hmC hotspots are rarely unmethylated but frequently associated with moderate levels of DNA methylation in the range of 20% to 50% (Figure 1e), which is consistent with significant but incomplete demethylation activity occurring at the majority of 5hmC hotspots. This observation is also supported by a recent report describing enrichment of 5hmC and enhancer activity in genomic regions with intermediate DNA methylation [42]. Finally, we use EpiExplorer to perform a Gene Ontology analysis for those genes that are located in close vicinity of 5hmC hotspots (Figure 1f). The 5hmC-associated genes are enriched for specific annotation terms related to gene regulation and development, including 'regulation of signal transduction', 'cell differentiation' and 'anatomical structure morphogenesis'.

Taken together, these EpiExplorer analyses suggest testable hypotheses about the role of 5hmC in human ES cells. For example, active DNA demethylation - with 5hmC as an intermediate - may protect developmental enhancers from gaining DNA methylation in undifferentiated cells. This mechanism may help ES cells retain their developmental potential in the presence of high levels of DNA methyltransferase activity. In addition, active DNA methylation could help avoid the accumulation of cancer-associated epigenetic alterations in undifferentiated cells, given that the sites of such alterations frequently overlap with developmental regulator elements [43]. To provide further support for these hypotheses, we can export the analyzed data from EpiExplorer to the Genomic HyperBrowser and perform more rigorous statistical testing than is possible within EpiExplorer. And most importantly, it will be necessary to confirm biological significance by in-depth functional dissection of the interplay between 5hmC and H3K4me1 at developmental enhancers. Such wet-lab studies are laborious to conduct and inherently limited to a small number of candidate genes or genomic regions, thus requiring careful selection of the most relevant candidates. EpiExplorer can help guide the selection of suitable regions for functional follow-up, as illustrated in the following case study.

### Interactive identification and prioritization of candidate regions using EpiExplorer

**Figure 2:**
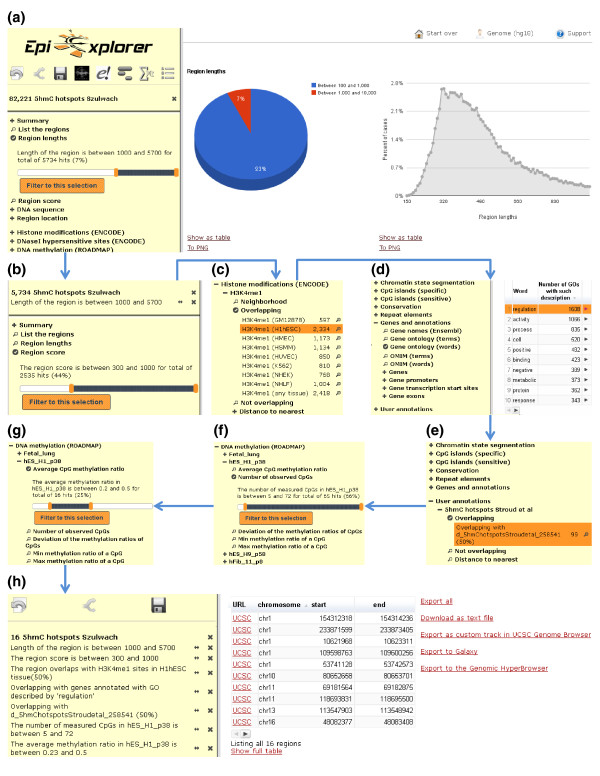
**Dynamic filtering of epigenome data identifies candidate regions for further analysis**. Using successive filtering steps, a genomic dataset with 82,221 hotspots of 5-hydroxymethylcytosine (5hmC) in human ES cells [35] is refined to a list of 16 regions that provide strong candidates for investigating the functional association between 5hmC and H3K4me1-marked enhancer elements. **(a) **Filtering with a minimum length threshold of 1 kb yields 5,734 genomic regions. **(b) **Filtering with a minimum 5hmC hotspot score threshold of 300, which corresponds to a detection significance of 10^-30 ^or better, yields 2,535 genomic regions. **(c) **Filtering for overlap with H3K4me1 peaks in a human ES cell line (H1hESC) yields 2,334 genomic regions. **(d) **Filtering for association with genes that are annotated with any of the 1,608 Gene Ontology terms containing the word 'regulation' yields 1,064 genomic regions. **(e) **Filtering for overlap with an alternative dataset of 5hmC hotspots [44] yields 99 genomic regions. **(f) **Filtering for a minimum DNA methylation coverage threshold of five CpGs yields 65 genomic regions. **(g) **Filtering for intermediate DNA methylation with levels in the range of 20% to 50% yields 16 genomic regions. **(h) **EpiExplorer screenshot showing the final list of candidate regions, ready for visualization in a genome browser, for download and manual inspection, and for export to other web-based tools for further analysis.

When studying mechanisms of gene regulation, it is often necessary to select a few model genes or genomic regions for a more detailed investigation than is possible with genome-wide methods. Good candidates should be informative of the phenotype of interest but must also be easily tractable experimentally. EpiExplorer is a powerful tool for identifying such candidates through several steps of region set filtering and interactive refinement of the selection criteria. For example, to unravel the mechanistic basis of the association between 5hmC and H3K4me1-marked enhancer elements (as described in the previous section) we need to identify a handful of strong examples for this kind of association, which can then be studied using biochemical and molecular biological assays. Good candidate regions should exhibit robust enrichment for both 5hmC and H3K4me1, proximity to genes involved in transcriptional regulation, and moderate levels of DNA methylation. With EpiExplorer, it is straightforward to distill such candidate regions from the complete list of 82,221 5hmC hotspots (Figure 2).

First, we inspect the length distribution of 5hmC hotspots (Figure 2a) and retain only those hotspots with a minimum length of 1 kb, which removes spurious peaks that are occasionally introduced by short repetitive elements in the genomic DNA sequence. Second, we filter for a detection significance of 10^-30 ^or better in order to focus the analysis on the most clear-cut 5hmC hotspots (Figure 2b). Third, we require evidence of an enhancer-associated chromatin signature and retain only those 5hmC hotspots that overlap with H3K4me1 peaks (Figure 2c). Fourth, in order to maximize relevance of the candidate regions for drawing conclusions about gene regulation, we restrict the analysis to genomic regions located in the vicinity of genes that are annotated with Gene Ontology terms containing the word 'regulation' (Figure 2d). Fifth, we import an additional dataset of 5hmC hotspots in human ES cells [44] into EpiExplorer and retain only those hotspots that are present in both datasets (Figure 2e). Because these two 5hmC datasets were obtained using different experimental methods, our selection of consensus hotspots should effectively remove technical artifacts of either dataset. Sixth, to be able to robustly select 5hmC hotspots with intermediate DNA methylation levels in the last step, we discard those regions for which insufficient bisulfite sequencing coverage is available from the Roadmap Epigenomics datasets (Figure 2f). Seventh and last, we focus the analysis on those regions that exhibit moderate levels of DNA methylation because it is plausible to hypothesize that the epigenetic state of these regions might be the result of significant but incomplete levels of active DNA demethylation (Figure 2g). Each of these filtering steps is interactively performed using EpiExplorer. Together they bring the original list of 82,221 5hmC hotspots down to 16 regions that fulfill all criteria and constitute strong candidates for a mechanistic study exploring the association between 5hmC and H3K4me1-marked enhancer elements (Figure 2h).

To facilitate follow-up research, EpiExplorer provides extensive functionality for data export and visualization using external tools. First, every genomic region set in EpiExplorer can be exported and visualized as a custom track in the UCSC Genome Browser [11], which is usually a good starting point for designing locus-specific experiments. Second, the results generated by EpiExplorer can be transferred to Galaxy [18] in order to perform sequence motif search, primer design and a number of other useful analyses that facilitate wet-lab experimental planning. Third, export to the Genomic HyperBrowser [20] can provide the starting point for additional statistical analyses performed online. Fourth, it is possible to export and download all region sets as text files for customized analysis with spreadsheet software (for example, Excel) or statistical analysis tools (for example, R).

## Concepts and algorithms

EpiExplorer's distinguishing feature is the ability to perform a broad range of genome-scale analyses in seconds, thus enabling live exploration, visualization, summarization and interactive filtering of large genomic datasets. Our use of multiple filtering and iterative refinement has important similarities with the concept of faceted search, which is a widely studied paradigm in information retrieval [45,46]. It critically depends on the speed with which complex search queries can be handled. In EpiExplorer, we achieve the necessary runtime performance by using the CompleteSearch engine [47], which has originally been developed for semi-structured text search in large document repositories. Through creative use of prefix indexing, CompleteSearch provides native support for advanced search features such as query autocompletion and database-style JOIN operations, and it has been shown to outperform more standard approaches based on inverted indices [47]. As a result, EpiExplorer was able to complete more than 95% of approximately 4,000 genome-scale analyses performed in the context of the 5hmC case studies in less than two seconds (Table 1).

**Table 1:** EpiExplorer's response time and memory footprint across thousands of actual user analyses

	Putative enhancers	CpG islands (specific)	Transcription start sites	Gene promoters (-5kb to 1kb)	**5hmC hotspots (Szulwach **[35]**)**	Genome-wide tiling regions (5kb)
Number of genomic regions	1,762	27,638	36,655	36,655	82,221	616,093
Preprocessing time (h)	0.2	0.8	0.9	0.9	1.5	17
Search index size (MB)	11	145	122	127	240	962
Mean query time (s)	0.02	0.06	0.12	0.13	0.2	0.8
95th percentile query time (s)	0.07	0.34	0.5	0.57	0.64	3.2
Percent queries completed in ≤2 s	100%	99.9%	99.7%	99.1%	99.1%	88%

In order to utilize these powerful text search operations for genomic analyses, we developed an encoding scheme that translates heterogeneous genome and epigenome datasets into a semi-structured text format (Figure S2 in Additional file 1). Each genomic region (such as a CpG island or 5hmC hotspot) is represented by a text document containing keywords for all its annotation features; and we use CompleteSearch to create a search index for the collection of text documents representing the regions of each user-uploaded dataset. EpiExplorer keywords are structured hierarchically, which enables efficient analyses through prefix search at various levels of granularity. For example, the term *overlap:histones:H3K4me3 *selects all regions that overlap with an H3K4me3 peak in any tissue, while the more specific term *overlap:histones:H3K4me3:H1hESC *selects only those regions that overlap with an H3K4me3 peak in ES cells. Furthermore, we can perform autocompletion queries such as overlap:histones:H3K4me3:*, which returns the number of regions that overlap with an H3K4me3 peak separately for each tissue. EpiExplorer also encodes various numeric scores (such as overlap ratios and DNA methylation levels), which are specifically encoded for prefix text search as described in the Materials and methods section. Overall, the use of the CompleteSearch engine for semi-structured text search confers a level of flexibility, efficiency and scalability that would not be easy to achieve with a simple text-tagging approach or with a relational database management system. And despite our extensive reliance on text search, the user never has to formulate any textual search phrases - they are dynamically constructed based on the user interaction with EpiExplorer's graphical frontend.

**Figure 3:**
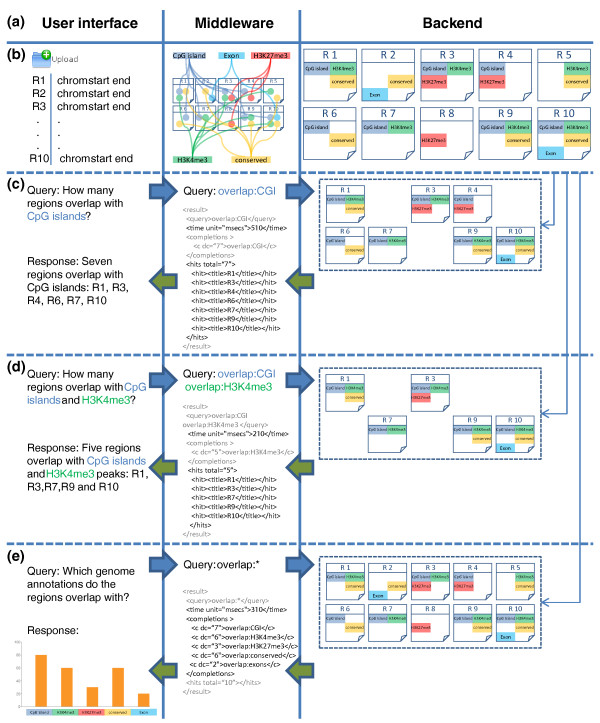
**Efficient text search enables live exploration of genome-scale datasets**. For three simple queries performed on a small set of genomic regions, this figure illustrates how EpiExplorer analyses are translated into text search queries, how these queries are run against a text index built from genomic data, how the responses are translated back into genome analysis results, and how the results are visualized in the user's web browser. **(a) **EpiExplorer's software architecture consists of three tiers: a web-based user interface, a middleware that translates between genomic analyses and text search queries, and a backend that efficiently retrieves matching regions for each query. **(b) **When a user uploads a genomic region set (here: chromosome, start and end position for ten regions named R1 to R10), the middleware annotates this region set with genome and epigenome data, encodes the results in a semi-structured text format, and launches a CompleteSearch server instance to host the corresponding search index. **(c) **To identify which regions overlap with a CpG island, a simple query *overlap:CGI *is sent to the backend, and the backend returns an XML file with the matching regions. **(d) **To identify regions that overlap with CpG islands as well as with H3K4me3 peaks, an AND search is performed (query: *overlap:CGI overlap:H3K4me3*), and the backend returns only regions that are annotated with both keywords. **(e) **To efficiently generate percent overlap diagrams, a prefix query overlap:* is sent to the backend, which identifies all possible completions of the prefix and returns the total number of regions matching each query completion.

Figure 3 illustrates the computational workflow of typical EpiExplorer analyses. Once a user-defined region set has been uploaded, the middleware annotates each genomic region with data from EpiExplorer's genome and epigenome annotation database, encodes these annotations as structured text and creates a dedicated CompleteSearch instance supporting search on this region set. For every analysis that is requested via the user interface, EpiExplorer's middleware constructs a text search query that is then sent to the corresponding CompleteSearch instance. The text search engine runs the query against its index and returns a set of matching regions. The middleware decodes the textual format and passes the results on to the user interface, which visualizes the data in ways that facilitate intuitive exploration of genomic datasets (Figures 1 and 2; Figure S1 in Additional file 1). This computational approach makes it possible to solve complex non-textual analysis problems using single queries to a text search index, and thereby it enables the live exploration of large genomic datasets.

## Discussion

Toward the goal of interactively exploring large epigenomic datasets, EpiExplorer borrows key concepts from interactive web search. In contrast to genome browsers, which implement browse-and-zoom navigation similar to that of map viewing software, EpiExplorer was inspired by the interactive filter-and-refine workflow of web search engines: Most web searches start broadly (for example, with the search term 'epigenetics') and are then refined iteratively (for example, with the additional terms 'bioinformatic', 'software' and 'tools') until relevant websites show up among the top hits. EpiExplorer supports the same kind of exploratory search by making it easy to dynamically filter genomic region sets and by providing instant feedback in the form of graphical results summaries. Just like web search engines EpiExplorer is highly fault-tolerant, and it allows users to change any aspect of an analysis (for example, thresholds or filtering criteria) at any time without having to repeat previous steps.

The interactive nature of such analyses depends on fast response times, as any delay tends to stifle the creative act of live data exploration. For this reason, we designed and optimized EpiExplorer to complete complex genome-wide analyses in seconds, rather than the minutes or hours that are the norm for existing genome analysis toolkits (for example, Galaxy [18], Genomic HyperBrowser [20] and EpiGRAPH [22]). This level of runtime performance was achieved by utilizing an indexing algorithm that was originally developed for text search; and we anticipate that this design principle of EpiExplorer - to encode complex analyses into ultrafast text search queries - will be broadly applicable for interactive analysis of biomedical datasets (for example, for annotating disease-associated genotypes and in the interpretation of personal genomes).

Importantly, EpiExplorer is closely interconnected with other web-based tools, which allowed us to focus EpiExplorer on data exploration and hypothesis generation while relocating data preprocessing and in-depth statistical analysis to specialized tools such as Cistrome [23] and the Genomic HyperBrowser [20]. We illustrated EpiExplorer's utility for interactive data exploration by a case study of hydroxymethylation in relation to public reference epigenome datasets, which recreates and extends results from a recently published paper [35] in ten minutes of analysis time (detailed tutorials are available from the supplementary website [27]). With this example in mind, we are optimistic that EpiExplorer will help bridge the 'digital divide' in biomedical research and constitute a step toward making large-scale epigenome datasets more useful and readily explorable for researchers with little or no bioinformatic experience.

## Materials and methods

### Software architecture

EpiExplorer is implemented according to a three-tier architecture scheme (Figure S3 in Additional file 1). The web-based user interface communicates with EpiExplorer's middleware, which in turn is supported by an annotation database and dynamically loaded text search engines in the backend. The web-based interface enables users to explore, upload and refine genomic region datasets. The interface is highly dynamic through the combination of server-side scripting (in PHP) and client-side scripting (in JavaScript). EpiExplorer utilizes the jQuery library [48] for implementing flexible client-side interface functionality and Google Chart Tools [49] for generating interactive visualizations of the data. (The charts used by EpiExplorer do not exchange any data with Google or other servers and therefore do not compromise data privacy in any way.) All visualizations are dynamically generated based on region set data obtained via an XML-RPC connection with the middleware. The EpiExplorer middleware layer is implemented in the Python programming language and has two separate components. First, the annotation mapping module uses BEDTools [16] in combination with an annotation database (in the backend) to annotate user-uploaded datasets with genome and epigenome data. These annotations are translated into a semi-structured text format (Figure S2 in Additional file 1), and a text index is generated for each region set. the resulting text index is hosted by an instance of the CompleteSearch engine [47]. Second, the middleware's query processing module receives analysis requests from the web frontend, translates them into text search queries and polls the CompleteSearch instance that hosts the corresponding genomic region set. The CompleteSearch engine returns the results to the middleware, which decodes the text format and sends the results back to the user interface for visualization.

### Textual encoding of binary and categorical genomic attributes

EpiExplorer internally represents each genomic region as a text file that encodes region-specific annotations in a semi-structured text format. For binary and categorical attributes (such as a region's association with an H3K4me1 peak or a 5hmC hotspot), the key concept is overlap. Two genomic regions are treated as overlapping if they have at least one base pair in common, and it is often plausible to assume that region sets that overlap more frequently than expected by chance are involved in similar biological processes (for example, co-binding of functionally related transcription factors). To effectively handle such data in the context of text search, we define the prefix *overlap: *followed by an annotation identifier. For example, the word *overlap:genes *indicates that the current region overlaps with the body of a gene, *overlap:conserved *encodes the overlap with a conserved element, and *overlap:CGI *denotes overlap with a CpG island. Using CompleteSearch's prefix search functionality, it we can efficiently retrieve all completions of a given prefix. For example, the query overlap:* retrieves all possible completions of the prefix *overlap:*, reporting the number of regions for each completion (see Figure 3 for an example). In this way, overlap information for a large number of genome and epigenome attributes can be obtained via a single text search query that is almost always answered within seconds (Table 1). Furthermore, the general overlap query overlap:* can be refined according to the hierarchical structure of the encoding scheme. For example, the word overlap:histones:H3K4me3:* retrieves an overlap summary of the H3K4me3 mark for all cell types included in EpiExplorer, whereas *overlap:histones:H3K4me3:ES *only obtains the regions that overlap with an H3K4me3 peak in ES cells.

### Textual encoding of numeric genomic attributes

Many genomic attributes are numeric - for example, the CpG content or the distance to a neighboring gene. To be able to perform efficient text search on these attributes, we limit their numerical precision (number of digits) to a fixed number and use a binning scheme when necessary. We can then incorporate numeric score values into the textual encoding scheme by creating words such as *dnaseq:freq:CG:010*, which indicates that a genomic region exhibits a CpG frequency of 0.010 (1.0%). This textual encoding allows EpiExplorer to retrieve the distribution of CpG frequencies in a set of regions using the prefix query dnaseq:freq:CG:*, which facilitates the efficient plotting of histograms. Using CompleteSearch's *range *query feature, it is also straightforward to obtain all genomic regions with numeric attributes that fall into a certain range. For example, the query *dnaseq:freq:CG:010--dnaseq:freq:CG:050 *retrieves only those regions that have a CpG frequency of at least 1% and not more than 5%. Beyond region score attributes, additional numeric attributes supported by EpiExplorer include overlap ratios for filtering on the percent overlap between genomic regions as well as distances to neighboring genomic elements, which enable filtering steps such as 'identify all regions within 20 kb from the nearest gene'. Binary, categorical and numeric queries can be combined and iteratively refined in arbitrary ways. For example, the query *overlap:CGI dnaseq:freq:CG:010--dnaseq:freq:CG:050 *retrieves all regions that overlap with CpG islands and exhibit a CpG frequency in the range of 1% to 5%.

### Integration of gene-centric textual annotations

In addition to binary, categorical and numeric attributes, EpiExplorer also incorporates textual information that is associated with genes, which includes Gene Ontology terms and OMIM phenotypes. As these annotations are already in text format, they can be used directly as keywords in the text search index. However, because these textual annotations can be lengthy and often apply to multiple genomic regions overlapping with the same gene, it is not ideal to store them directly in the description of each region. Instead, EpiExplorer maintains genes and their textual annotations as separate documents and stores only the gene identifier in the annotation of every overlapping genomic region. For example, if a region overlaps with the *BRCA2 *gene, EpiExplorer will add the word *gene:BRCA2 *to the document that represents the region, while the lengthy textual annotations of BRCA2 are stored in a separate document named *gene:BRCA2*. To answer text search queries that include these gene annotations, EpiExplorer makes use of the database JOIN feature that is supported by CompleteSearch. This way, the results from a region-based search and the results from a gene-based search can be combined in a single query, and only the matches are returned for visualization.

### Dynamic visualization of search results and region sets

EpiExplorer visualizes the results of a text search using five types of dynamically generated diagrams.

#### The bar chart

The bar chart (see Figure 1a for an example) reports the percentage overlap of a selected region set with genomic regions of different types. Using the EpiExplorer control menu, it is straightforward to restrict a region set to those regions that overlap (or do not overlap) with another type of genomic regions shown in this diagram.

#### The area chart

The area chart (see Figure 1e for an example) is essentially a histogram, which summarizes the distribution of numeric attributes with a relatively narrow value range. The control menu provides a dynamic slider that can be used to restrict the selection to a subset of regions within a user-specified value range.

#### The pie chart

The pie chart (see Figure 2a for an example) is shown in addition to the area chart to summarize the distribution of numeric attributes that may span a wide value range. In this case, clicking any segment of the pie chart opens a zoomed-in area chart specific for the genomic regions that fall into the selected value range.

#### The neighborhood chart

The neighborhood chart (see Figure 1c for an example) illustrates the distribution of genome-wide maps - such as histone marks and transcription factor binding sites - in the vicinity of the selected region set. Average levels of overlap are calculated over all genomic regions in the set.

#### The bubble chart

The bubble chart (see Figure S1B in Additional file 1 for an example) plots the percentage of genomic regions that overlap with a given annotation (y-axis) against the total genome coverage of this type of annotation (x-axis). In this context, the genome coverage provides an indication of the expected overlap, highlighting annotations with substantially different overlap percentages. When used in comparison mode, an additional dimension is added to the bubble chart to represent the overlap of the annotations with the control set.

#### The enrichment chart

The enrichment chart (see Figure 1f for an example) summarizes gene-centric textual information in the form of a table and a word cloud. In the word cloud, the font size is scaled by the enrichment ratio, which is calculated relative to random expectation. Clicking on any annotation term refines the search to include only those regions that are associated with a gene carrying the corresponding annotation.

### Annotation of genomic region sets

EpiExplorer makes no conceptual distinction between default and user-uploaded region sets. Every feature that is available for default region sets can also be used on custom data. Upon upload, new region sets are automatically annotated with a broad range of genome and epigenome attributes that are maintained in EpiExplorer's annotation database (see Table S1 in Additional file 2 for a complete list). The user can also select custom region sets as annotations for other user-uploaded region sets. The current version of EpiExplorer provides full support for the human genome assemblies hg18/NCBI36 and hg19/GRCh37, as well as for the mouse genome assembly mm9/NCBIM37. By default, EpiExplorer annotates every region with its chromosomal position, region length, strand and score attributes (if included in the uploaded BED file), and with annotations of ten different types: DNA sequence composition, histone modifications, transcription factor binding sites, DNaseI hypersensitive sites, DNA methylation, chromatin state segmentation, CpG islands, evolutionary conservation, repeat elements and gene-associated attributes. These annotations are derived from the following sources: (i) DNA sequence composition attributes are calculated directly from the genomic DNA sequence, which was downloaded from the UCSC Genome Browser [11]. (ii) Histone modification data have been generated as part of the ENCODE project [6] and were obtained from the UCSC Genome Browser [50]. We used preprocessed peak regions for 11 histone modifications and chromatin marks (H3K4me1, H3K4me2, H3K4me3, H3K9ac, H3K9me1, H3K27ac, H3K27me3, H3K36me3, H4K20me1, CTCF and Pol2) in nine cell lines (GM12878, H1hESC, HepG2, HMEC, HSMM, HUVEC, K562, NHEK and NHLF; described in more detail in the ENCODE documentation [51]. (iii) Experimental data for transcription factor binding have also been generated as part of the ENCODE project and were obtained from the UCSC Genome Browser. We used preprocessed peaks for 33 transcription factors (AP2alpha, AP2gamma, ATF3, BDP1, BRF1, BRF2, cFos, cJun, cMyc, E2F1, E2F4, E2F6, GATA1, GATA2, GTF2B, HELFe, junD, MAX, NFE2, NFKB, Pol2, Pol3, Rad21, RPC155, SETDB1, SIRT6, TFIIIC110, TR4, XRCC4, YY1, ZNF263, ZNF274 and ZZZ3) in at least one cell line. (iv) DNA methylation data have been generated and preprocessed in the context of the Roadmap Epigenomics initiative [52] as described previously [53,54]. They include ten tissue types: ES cells, fetal brain, fetal heart, fetal kidney, fetal lung, fibroblasts, hematopoietic progenitor cells, skeletal muscle, smooth muscle and stomach mucosa. (v) Chromatin segmentation data were obtained from a recent paper describing a hidden Markov model segmentation of histone modification data from the ENCODE project [39]. (vi) DNaseI hypersensitive sites were also obtained from the ENCODE project. (vii) CpG island annotations were downloaded from the UCSC Genome Browser ('CpG islands (specific)') and from the CgiHunter website ('CpG islands (sensitive)') [55]. (viii) Evolutionary conservation data were obtained from the phastCons annotation track of the UCSC Genome Browser [56]. (ix) Repeat element annotations were obtained from the RepeatMasker annotation track in the UCSC Genome Browser [57]. (x) Gene-associated attributes were retrieved via Ensembl Biomart [58] and include the gene name, textual description as well as annotations from the Gene Ontology [24] and OMIM [25] databases.

### Advanced features

EpiExplorer provides a number of advanced features that are not essential for first-time users but can provide substantial added value when using EpiExplorer routinely for exploring genome and epigenome datasets.

#### Comparative analysis

To assess whether the association between a region set and an annotation attribute is biologically relevant, it is often helpful to repeat the comparison for a randomized control set. Such control sets are automatically generated when custom region sets are uploaded into EpiExplorer, simply by reshuffling the genomic position of all regions in the dataset. In addition, the user can select any region set that is available within EpiExplorer for use as a control set. Once a control set has been selected, it is automatically included as a reference (in grey) in all bar and area charts. Although the control set functionality does not replace statistical testing in a strict sense (which can be performed via EpiExplorer's export function to the Genomic HyperBrowser as illustrated in the corresponding tutorial on the supplementary website [27]), this feature is often informative for exploratory research because it provides the user with a visual intuition of the strength of association between genomic attributes.

#### Flexible OR refinements

While the combination of search terms with AND is considered standard for search engines, the CompleteSearch engine also supports OR queries. This feature gives the user additional flexibility for performing complex combinations of analyses. For example, the query *dnameth:ES:ratio:00--dnameth:ES:ratio:33|dnameth:ES:ratio:66--dnameth:ES:ratio:99 *selects all regions that are mostly unmethylated or mostly methylated.

#### Sharing results

EpiExplorer was developed with the paradigms of reproducible research in mind [59], and it provides several ways of documenting an analysis. Each user-uploaded region set is assigned a unique identifier that also serves as a password for accessing this dataset. Sharing this identifier with other researchers enables them to analyze the same dataset in EpiExplorer without any need for copying or transferring datasets. Furthermore, at any point in an EpiExplorer analysis, an identifying URL can be obtained that dynamically recreates the analysis and allows the user to follow up on the results without affecting the original analysis snapshot. Because all steps of an EpiExplorer analysis are documented in the control menu, the snapshot functionality ensures that EpiExplorer analyses are readily reproducible. This point is illustrated by the tutorials on the supplementary website [27], which provide a URL for each step of the analysis that automatically recreates the results when pasted into a web browser. EpiExplorer also supports the export of any region set as a downloadable BED file, its visualization as custom tracks in the UCSC Genome Browser and Ensembl, and the transfer into Galaxy and Galaxy-powered tools such as the Genomic HyperBrowser for further analysis; and it provides lists of gene identifiers for export to gene set tools such as DAVID and Gene Set Enrichment Analysis (GSEA). Every custom dataset, refinement and visualization is accessible only to its creator (unless explicitly shared with other researchers) and protected by strong identifiers functioning as passwords, thus ensuring the privacy of data and analyses. More information on export functionality, data sharing and confidentiality are provided in the tutorials on the supplementary website [27].

### Performance evaluation

EpiExplorer was designed for performance, in order to enable interactive exploration of large genome and epigenome datasets. Table 1 underlines this point by summarizing EpiExplorer's runtime performance and resource consumption for its five default region sets as well as for the user-uploaded set of 5hmC hotspots. The preprocessing time needed to annotate and index user-uploaded datasets is usually on the order of minutes to hours (depending on the size of the region set); but it has to be performed only once when a genomic region set is first uploaded into EpiExplorer, and the user can activate e-mail notification and/or actively check for progress of the calculation. The size of the resulting index structure is typically on the order of few hundred megabytes. Once an index structure has been created, it takes very limited resources for the EpiExplorer server to perform analyses on the corresponding region set. We evaluated the performance of EpiExplorer by measuring the CompleteSearch response times on thousands of queries that were run during the preparation of this paper. For every region set, we measured the average query time, the time in which 95% of queries were processed, and the percentage of queries that required less than 2 seconds (Table 1). The results show that the average query time for each region set is consistently below 1 second, and that 95% of all analyses even for the largest region set completed in less than 4 seconds, which makes the dynamic exploration of datasets via EpiExplorer a continuous and interactive process for the users.

### Scalability

To be able to handle the wave of epigenome data that are being produced by international consortia, EpiExplorer was designed to scale to high user load and to be readily extensible with additional datasets. Because of the parallel nature of the computation-heavy backend, performance bottlenecks resulting from increasing user load can be resolved simply by adding more compute nodes for the backend. Furthermore, due to dynamic loading of backend instances, only parts of the indices of those region sets that are actively used need to be kept in memory, while additional user datasets are quickly reloaded from hard disk when a user accesses them. In its current version, EpiExplorer already handles hundreds of genome and epigenome annotations (Table S1 in Additional file 2) and hundreds of custom datasets, even though we are not currently utilizing all the parallelization options that the EpiExplorer architecture provides.

### Extensibility

Incorporating new datasets into EpiExplorer is straightforward and can be done by any user, provided that the data are available in (or can be converted to) one of several supported data types, namely genomic regions with or without a quantitative score and optionally including additional annotations such as strand information. For example, adding a new histone modification requires just a few mouse clicks in the frontend and less than an hour of computation time for the middleware and backend. Adding support for new genome assemblies is also relatively straightforward though not fully automated, as it requires minor modifications of the frontend and middleware. Finally, the textual encoding behind EpiExplorer is flexible enough to incorporate conceptually new data types (for example, three-dimensional genomic interaction maps that link two or more genomic regions together), which would require modifications in the middleware's annotation mapping component and the implementation of new diagram types (for example, Circos plots) in the frontend. The source code of EpiExplorer is freely available for download from the support menu on EpiExplorer's supplementary website [27].

### Supplementary website

The supplementary website [27] provides additional material describing in detail how EpiExplorer can be used to recreate all analyses described in this paper. Specifically, the website includes dataset identifiers for loading the 5hmC hotspots into EpiExplorer and slideshow tutorials that provide a general introduction into EpiExplorer as well as a step-by-step description of how Figure 1, Figure 2 and Figure S1 were created.

## Abbreviations

5hmC: 5-hydroxymethylcytosine; ES: embryonic stem; GO: Gene Ontology; OMIM: Online Mendelian Inheritance in Man.

## Competing interests

The authors declare that they have no competing interests.

## Authors' contributions

KH conceived the project with support from TL and CB. KH, TL and CB planned the research. KH and CB conducted the research. KH developed the methods and software. FA extended the annotation database and assisted with software development. HB contributed software, ideas and technical guidance. KH and CB wrote the manuscript with input from HB and TL. All authors have read and approved the manuscript for publication.

## Supplementary Material

Additional file 1 **Supplemental figures**. Click here for file

Additional file 2 **Table S1**. Click here for file
