# Supplementary material for: EpiExplorer: live exploration and global analysis of large epigenomic datasets
Source: Genome Biol. 2012 Oct 3;13(10):R96. doi: 10.1186/gb-2012-13-10-r96 (PMC3491424; doi:10.1186/gb-2012-13-10-r96)
Supplement: Additional file 1 — Supplemental figures. [file gb-2012-13-10-r96-S1.PDF]

Additional File 1

EpiExplorer: live exploration and global analysis of large epigenomic datasets

Konstantin Halachev, Hannah Bast, Felipe Albrecht, Thomas Lengauer, Christoph Bock

Inventory

Supplementary References

Figure S1. EpiExplorer readily rediscovers known epigenetic characteristics of CpG islands

Figure S2. EpiExplorer encodes genomic data in a format that is suitable for efficient text search

Figure S3. EpiExplorer implements a parallelized and scalable software architecture

Text S1. Case study of EpiExplorer applied to the epigenetic characterization of CpG islands

Supplementary References

Supplemental Figure Legends

Figure S1. EpiExplorer readily rediscovers known epigenetic characteristics of CpG islands

(A) Bar chart summarizing the percent overlap (y-axis) between CpG islands and various genomic datasets (x-axis) in H1hESC cells.
(B) Bubble chart plotting the percent overlap (y-axis) between CpG islands and H3K4me3 peaks in specific tissues (color-coded) against the total genomic coverage of all corresponding peaks (x-axis)
(C) Neighborhood plot illustrating the percent overlap (y-axis) with histone H3K4me3 peaks in the vicinity of CpG islands (x-axis). Line colors correspond to histone modification data for different cell types.

(D) Neighborhood plot illustrating the percent overlap (y-axis) with histone H3K27me3 peaks in the vicinity of CpG islands (x-axis). Line colors correspond to histone modification data for different cell types.

(E) Percent overlap (y-axis) of 13,519 CpG islands located within one kilobase from a gene transcription start site (orange) and 2,327 CpG islands located at least 20 kilobases from the nearest gene (gray) with genome and epigenome annotation data (x-axis)

(F) Percent overlap (y-axis) of 15,377 constitutively unmethylated CpG islands (orange, less than 30% methylation in seven tissues) and 3,171 constitutively methylated CpG islands (grey, more than 60% methylation in the same seven tissues) with genome and epigenome annotation data (x-axis).

(G) Overview of the length distribution of constitutively unmethylated CpG islands (left) and constitutively methylated CpG islands (right).

(H) Histogram illustrating the distribution of CpG dinucleotide frequencies among constitutively unmethylated CpG islands (orange) and among constitutively methylated CpG islands (grey).

(I) Histogram illustrating the distribution of TpG dinucleotide frequencies among constitutively unmethylated CpG islands (orange) and among constitutively methylated CpG islands (grey).

Figure S2. EpiExplorer encodes genomic data in a format that is suitable for efficient text search

Schematic outline of genomic regions encoded as text and represented in an efficient index structure. Genomic region files are uploaded to EpiExplorer in the UCSC Genome Browser's BED format [1], with mandatory col-

umns specifying the chromosome, start and end positions of each region (step 1). The EpiExplorer middleware annotates the uploaded regions with qualitative and quantitative attributes such as overlap with CpG islands and distance to the nearest CpG island (step 2). A text document is created for every genomic region, and its annotations are encoded in a semi-structured text format (step 3). To enable alphanumeric search for numeric attributes, heading zeros are added until all numbers have the same number of digits. Two sorted lists are created, one containing document identifiers and the other containing keyword identifiers (step 4). Finally, CompleteSearch creates a text index connecting sorted word identifier ranges with sorted document-word pairs, which handles text search queries in a highly efficient fashion (step 5).

Figure S3. EpiExplorer implements a parallelized and scalable software architecture

Schematic outline of EpiExplorer's software architecture, consisting of a web-based user interface, a query processing and annotation mapping middleware, and a text-search backend. The user interface is a dynamic web-based frontend implemented in PHP and JavaScript. The middleware is implemented in Python and translates between genomic analyses requested by the user interface and text search queries sent to the backend. It also performs annotation of user-uploaded datasets using BEDTools [2] and manages CompleteSearch engine instances that are running in the backend. The backend of EpiExplorer consists of an annotation database implemented using SQLite and a collection of CompleteSearch server instances (one for each region set) that respond to text search queries sent by the middleware. The backend can be parallelized across multiple servers to increase performance. Unused CompleteSearch instances are automatically suspended to disk, from where they can be reactivated with minimal delay.

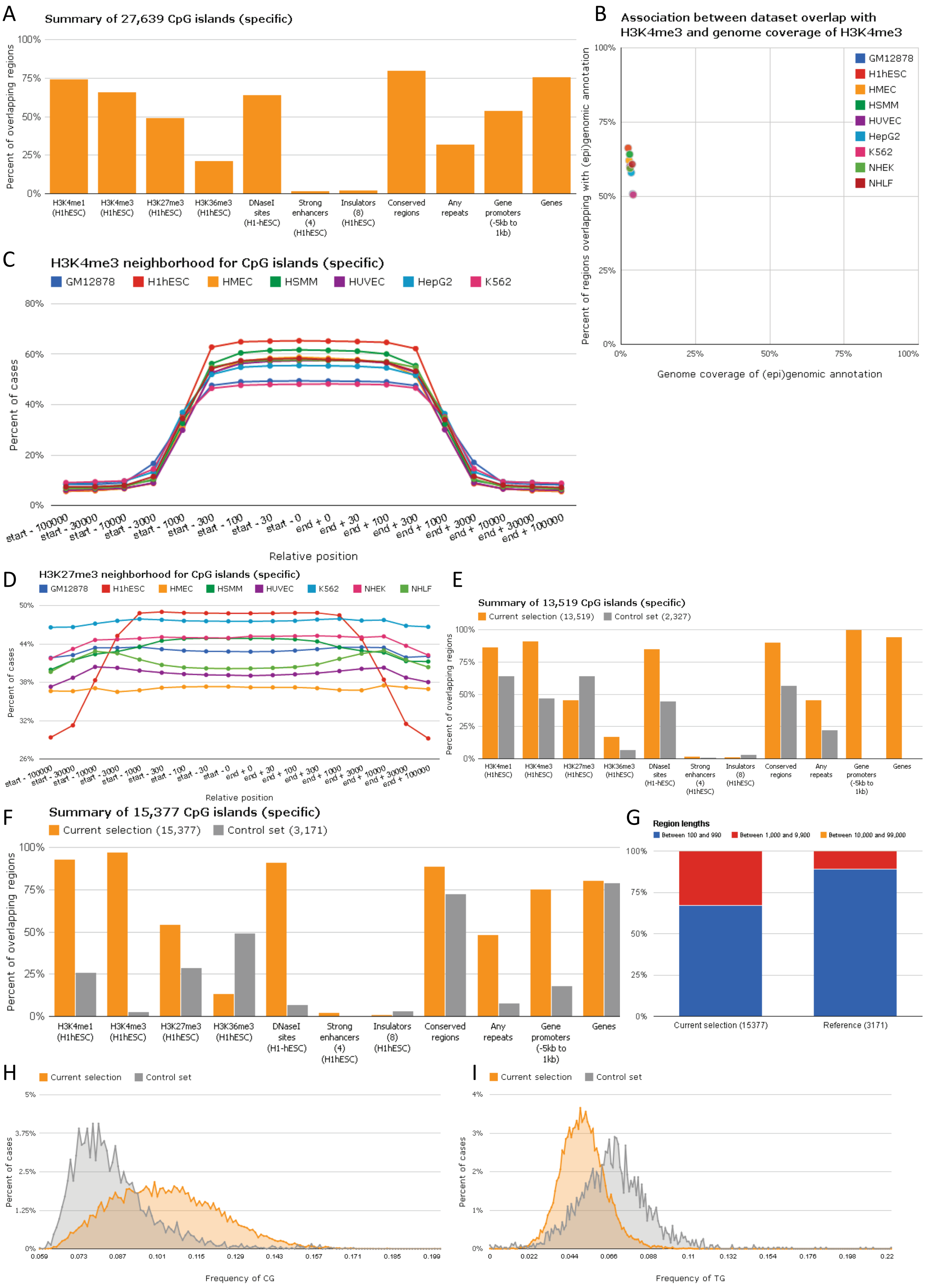

A
Summary of 27,639 CpG islands (specific)
Percent of overlapping regions
100%
75%
50%
25%
0%
H3K4me1 (H1hESC)
H3K4me3 (H1hESC)
H3K27me3 (H1hESC)
H3K36me3 (H1hESC)
DNaseI sites (H1-hESC)
Strong enhancers (4) (H1hESC)
Insulators (8) (H1hESC)
Conserved regions
Any repeats
Gene promoters (-5kb to 1kb)
Genes
B
Association between dataset overlap with H3K4me3 and genome coverage of H3K4me3
Percent of regions overlapping with (epi)genomic annotation
Genome coverage of (epi)genomic annotation
GM12878
H1hESC
HMEC
HSMM
HUVEC
HepG2
K562
NHEK
NHLF
C
H3K4me3 neighborhood for CpG islands (specific)
GM12878
H1hESC
HMEC
HSMM
HUVEC
HepG2
K562
80%
60%
40%
20%
0%
Percent of cases
start - 100000
start - 30000
start - 10000
start - 3000
start - 1000
start - 300
start - 100
start - 30
start - 0
end + 0
end + 30
end + 100
end + 300
end + 1000
end + 3000
end + 10000
end + 30000
end + 100000
Relative position
D
H3K27me3 neighborhood for CpG islands (specific)
GM12878
H1hESC
HMEC
HSMM
HUVEC
K562
NHEK
NHLF
50%
44%
38%
32%
26%
Percent of cases
Relative position
E
Summary of 13,519 CpG islands (specific)
Current selection (13,519)
Control set (2,327)
F
Summary of 15,377 CpG islands (specific)
Current selection (15,377)
Control set (3,171)
G
Region lengths
Between 100 and 990
Between 1,000 and 9,900
Between 10,000 and 99,000
Current selection (15377)
Reference (3171)
H
Current selection
Control set
5%
3.75%
2.5%
1.25%
0%
Percent of cases
0.059
0.073
0.087
0.101
0.115
0.129
0.143
0.157
0.171
0.185
0.199
Frequency of CG
I
Current selection
Control set
4%
3%
2%
1%
0%
Percent of cases
0
0.022
0.044
0.066
0.088
0.11
0.132
0.154
0.176
0.198
0.22
Frequency of TG

Step	Description	Representation
1. Upload	The user uploads a set of genomic regions (in standard BED format)	

region	chrom	start	end
Region 1	chr1	1000	4240
Region 2	chr2	500	1545
Region 3	chr1	8300	8850
Region 4	chr5	3100	3400

Step	Description	Representation
2. Annotate	Each genomic region is annotated with a broad range of genomic attributes	

Region	chrom	Length	Frequency of CpG	Overlap with CGI	Distance to nearest CGI
Region 1	chr1	3240	0.07	34%	0
Region 2	chr2	1045	0.02	0%	521
Region 3	chr1	550	0.05	5%	0
Region 4	chr5	300	0.16	80%	0

Step	Description	Representation
3. Convert to text	Every region is represented as a text document and its annotations are translated into words	

Region 1	Region 2	Region 3	Region 4
chr1<br>length:3240<br>frequency:CG:07<br>overlapratio:CGI:34<br>overlap:CGI	chr2<br>length:1045<br>frequency:CG:02<br>distanceTo:CGI:521	chr1<br>length:0550<br>frequency:CG:05<br>overlapratio:CGI:05<br>overlap:CGI	chr5<br>length:0300<br>frequency:CG:16<br>overlapratio:CGI:80<br>overlap:CGI

Step	Description	Representation
4. Sort	Words and documents are sorted & assigned unique identifiers	

Doc ID	Document
D1	Region 1
D2	Region 2
D3	Region 3
D4	Region 4

Word ID	Word	Word ID	Word
W1	chr1	W9	length:0300
W2	chr2	W10	length:0550
W3	chr5	W11	length:1045
W4	distanceTo:CGI:521	W12	length: 3240
W5	frequency:CG:02	W13	overlap:CGI
W6	frequency:CG:05	W14	overlapratio:CGI:05
W7	frequency:CG:07	W15	overlapratio:CGI:34
W8	frequency:CG:16	W16	overlapratio:CGI:80

Step	Description	Representation
5. Create index	Sorted lists are stored in memory such that blocks correspond to ranges of word IDs and contain all pairs of document/word IDs in a given range	

Block	Word ID range	Corresponding words	document-word pairs
B1	W1 - W3	chr1, chr2, chr5	(D1,W1) (D2,W2) (D3,W3) (D4,W1)
B2	W4 - W8	distanceTo:CGI:521, frequency:CG:02, frequency:CG:05, frequency:CG:07, frequency:CG:16	(D1,W7) (D2,W4) (D2,W5) (D3,W6) (D4,W8)
B3	W9 - W12	length:0300, length:0550, length:1045, length:3240	(D1,W13) (D1,W15) (D3,W13) (D3,W14) (D4,W13) (D4,W16)
B4	W13- W16	overlap:CGI, overlapratio:CGI:05, overlapratio:CGI:34, overlapratio:CGI:80	(D1,W13) (D1,W15) (D3,W13) (D3,W14) (D4,W13) (D4,W16)

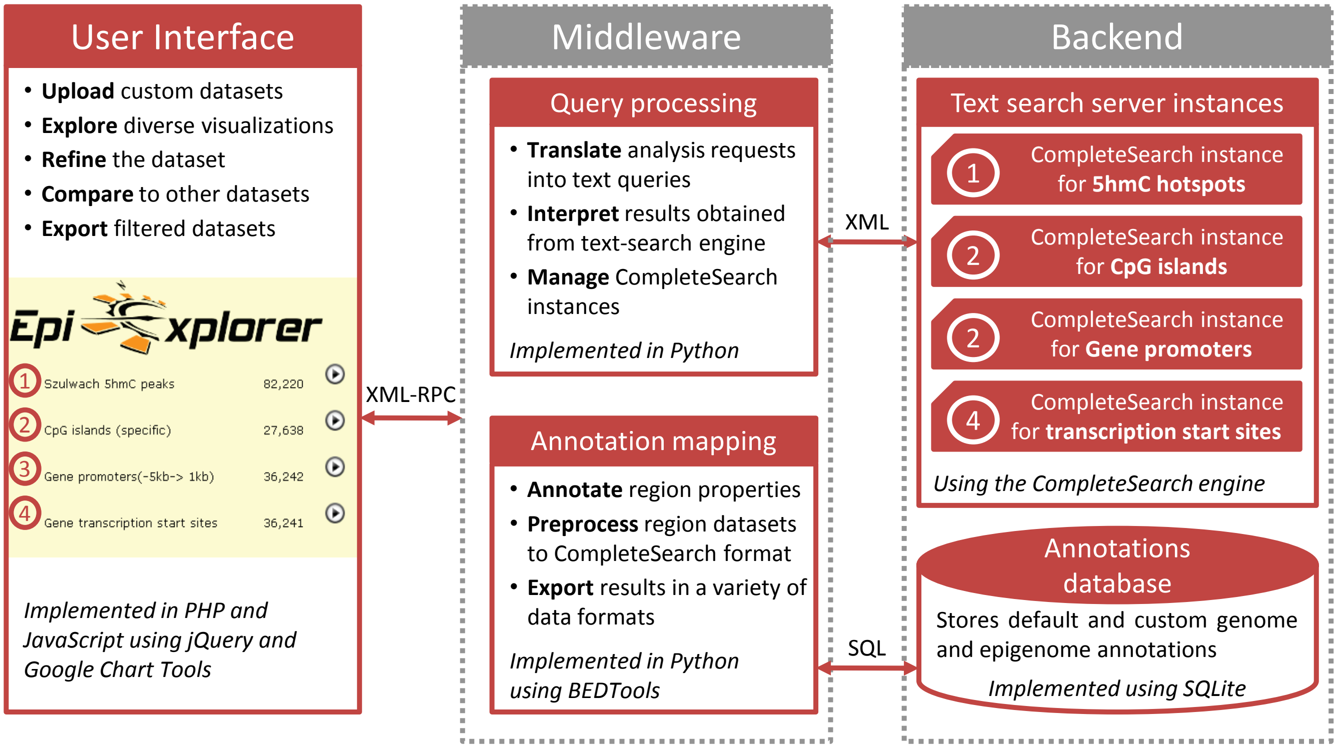
User Interface
• Upload custom datasets
• Explore diverse visualizations
• Refine the dataset
• Compare to other datasets
• Export filtered datasets
EpiExplorer
1 Szulwach 5hmC peaks 82,220
2 CpG islands (specific) 27,638
3 Gene promoters(-5kb-> 1kb) 36,242
4 Gene transcription start sites 36,241
Implemented in PHP and JavaScript using jQuery and Google Chart Tools
XML-RPC
Middleware
Query processing
• Translate analysis requests into text queries
• Interpret results obtained from text-search engine
• Manage CompleteSearch instances
Implemented in Python
Annotation mapping
• Annotate region properties
• Preprocess region datasets to CompleteSearch format
• Export results in a variety of data formats
Implemented in Python using BEDTools
XML
SQL
Backend
Text search server instances
1 CompleteSearch instance for 5hmC hotspots
2 CompleteSearch instance for CpG islands
2 CompleteSearch instance for Gene promoters
4 CompleteSearch instance for transcription start sites
Using the CompleteSearch engine
Annotations database
Stores default and custom genome and epigenome annotations
Implemented using SQLite

Supplementary Text

Text S1. Case study of EpiExplorer applied to the epigenetic characterization of CpG islands

CpG islands account for some of the most important regulatory regions in the human genome [3]. These regions exhibit highly non-random epigenetic characteristics: On the one hand, most CpG islands are enriched for histone modifications indicative of open chromatin (e.g., H3K4me1 and H3K4me3), and they exhibit low levels of DNA methylation. On the other hand, specific subsets of CpG islands have been described as highly methylated or enriched for the repressive histone modification H3K27me3 [4-9]. In order to validate EpiExplorer on the well-studied topic of epigenetic regulation at CpG islands, here we analyze the characteristics of CpG islands across the human genome, using EpiExplorer's functionality for exploring genomic region sets in the context of public genome and epigenome datasets.

CpG islands are already available as one of EpiExplorer's default region sets; hence it is not necessary to upload any new dataset to perform this analysis. Once we select "CpG islands (specific)" from the exploration menu on the left of EpiExplorer's start screen, EpiExplorer displays a summary of genome and epigenome annotations that CpG islands co-localize with (Figure S1A in Additional File 1). According to this diagram, more than half of all CpG islands overlap with Ensembl-annotated gene promoter regions, which is a well-established observation in the literature [10, 11]. Furthermore, we observe that two-thirds of all CpG islands overlap with the promoter-associated histone H3K4me3 mark in ES cells (Figure S1A in Additional File 1) and in other tissues (Figure S1B in Additional File 1). This observation underlines that a large subset of CpG islands indeed carry the key chromatin mark indicative of active promoters, and it constitutes a substantial enrichment as genomic regions carrying this mark cover only two to four percent of the genome (Figure S1B in Additional File 1). Furthermore, EpiExplorer's neighborhood plot (Figure S1C in Additional File 1) highlights how strongly and specifically the H3K4me3 mark is enriched at CpG islands compared to the broader genomic neighborhood of these regions.

While association with open chromatin appears to be the default state of most bona fide CpG islands in the human genome [4, 8], it has been shown that a subset of CpG islands are frequently associated with the repressive histone H3K27me3 mark [12, 13]. CpG islands have also even been reported to play a role in recruiting Polycomb proteins and the H3K27me3 mark in ES cells [7]. An EpiExplorer neighborhood plot shows specific and localized enrichment of the H3K27me3 mark in a human ES cell line, with an enrichment peak that ranges from one kilobase upstream of the annotated CpG island borders to one kilobase downstream (Figure S1D in Additional File 1). This ES-cell specific enrichment peak is only marginally broader than the one observed for the H3K4me3 mark (Figure S1C in Additional File 1). In contrast, for cell types other than ES cells, we observe elevated levels of H3K27me3 in a broad neighborhood surrounding CpG islands, consistent with the observation that localized peaks of H3K27me3 in ES cells are resolved into broad H3K27me3-enriched BLOCs in differentiated cells [14].

Despite the strong overlap of CpG islands with gene promoters and other genic regions (Figure S1A in Additional File 1), almost a quarter of CpG islands (6,705 in total) do not overlap with any annotated promoter regions or genes and are therefore categorized as intergenic CpG islands [15]. Some of these intergenic CpG islands may be linked to genes and promoters that are currently missed by genome annotations (e.g. lincRNAs), but others may have non-canonical roles for example as distal enhancers or as anchor points in the maintenance of three-dimensional genome organization. Using the refinement tools of EpiExplorer, we can dynamically reduce the set of all CpG islands to those that are clearly intergenic (i.e. located at least 20 kilobases distant from the nearest gene) and compare their properties to a set of promoter-associated CpG islands (i.e. located within a kilobase of an annotated transcription start site). The results show that intergenic CpG islands less frequently exhibit the promoter-associated H3K4me3 mark and the transcription-associated H3K36me3 mark than promoter-associated CpG islands (Figure S1E in Additional File 1), consistent with their intergenic nature. On the other hand, they are associated more frequently with H3K27me3 peaks and insulator elements (Figure S1E in Additional File 1), which is suggestive of a structural role in the organization of chromatin.

We also explored the distribution of DNA methylation among CpG islands. While most CpG islands appear to be unmethylated in the germline and thus protected from the increased C-to-T mutation rates associated with cytosine methylation [5, 16, 17], a subset of CpG islands becomes methylated during somatic tissue differentiation [18, 19]. Furthermore, certain types of repeat-associated and exonic CpG islands appear to be methylated in all tissues and retain their moderate levels of CpG density by means other than the absence of DNA methylation in the germline [5, 20]. To compare the genomic characteristics of methylated and unmethylated CpG islands, we derived within EpiExplorer a test set of constitutively unmethylated CpG islands and a reference set of constitutively methylated CpG islands (Figure S1F in Additional File 1). Comparison of both types (unmethylated CpG islands shown in orange, methylated ones in grey) identified striking enrichment for open-chromatin associated marks (H3K4me1, H3K4me3, DNaseI hypersensitive sites) among unmethylated CpG islands. In contrast, methylated CpG islands were strongly associated with the transcription-linked H3K36me3 mark and exhibited a similar level of evolutionary conservation and gene association as unmethylated CpG islands.

The characteristic differences between unmethylated and methylated CpG islands are not limited to their genomic location relative to genes and chromatin marks, but also include the genomic DNA sequence of the CpG islands themselves. Consistent with previous reports that identified high CpG island length and CpG density as strong predictors of low DNA methylation levels [8, 9, 16, 21], the EpiExplorer analysis shows that unmethylated CpG islands tend to be longer (Figure S1G in Additional File 1) and exhibit a CpG density distribution that is substantially shifted toward increased CpG densities compared to their methylated counterparts (Figure S1H in Additional File 1). In contrast, the TpG density distribution shows an opposite trend (Figure S1I in Additional File 1), supporting the notion that high levels of DNA methylation are directly linked to the accumulation of C-to-T mutations in the germline.

In summary, these observations suggest that CpG islands are regulated in different ways by three epigenetic marks, histone H3K4me3, histone H3K27me3 and DNA methylation. The presence of H3K4me3 is strongly correlated with low levels of DNA methylation. Furthermore, H3K27me3 overlaps with H3K4me3 at a subset of CpG islands (in particular for ES cells), while co-localization between H3K27me3 and DNA methylation is rare and only observed at CpG islands that are not particularly CpG-rich. All of these results are easy to verify using EpiExplorer, as described in a step-by-step tutorial on the Supplementary Website [22].

Supplemental References

1. **UCSC Genome Browser BED format documentation** [http://genome.ucsc.edu/FAQ/FAQformat.html#format1]
2. Quinlan AR, Hall IM: **BEDTools: a flexible suite of utilities for comparing genomic features**. *Bioinformatics* 2010, **26**:841-842.
3. Deaton AM, Bird A: **CpG islands and the regulation of transcription**. *Genes Dev* 2011, **25**:1010-1022.
4. Bock C, Walter J, Paulsen M, Lengauer T: **CpG island mapping by epigenome prediction**. *PLoS Comput Biol* 2007, **3**:e110.
5. Cohen NM, Kenigsberg E, Tanay A: **Primate CpG islands are maintained by heterogeneous evolutionary regimes involving minimal selection**. *Cell* 2011, **145**:773-786.
6. ENCODE Project Consortium: **Identification and analysis of functional elements in 1% of the human genome by the ENCODE pilot project**. *Nature* 2007, **447**:799-816.
7. Mendenhall EM, Koche RP, Truong T, Zhou VW, Issac B, Chi AS, Ku M, Bernstein BE: **GC-rich sequence elements recruit PRC2 in mammalian ES cells**. *PLoS Genet* 2010, **6**:e1001244.
8. Straussman R, Nejman D, Roberts D, Steinfeld I, Blum B, Benvenisty N, Simon I, Yakhini Z, Cedar H: **Developmental programming of CpG island methylation profiles in the human genome**. *Nat Struct Mol Biol* 2009, **16**:564-571.
9. Weber M, Hellmann I, Stadler MB, Ramos L, Pääbo S, Rebhan M, Schübeler D: **Distribution, silencing potential and evolutionary impact of promoter DNA methylation in the human genome**. *Nat Genet* 2007, **39**:457-466.
10. Bajic VB, Tan SL, Christoffels A, Schönbach C, Lipovich L, Yang L, Hofmann O, Kruger A, Hide W, Kai C, Kawai J, Hume DA, Carninci P, Hayashizaki Y: **Mice and men: their promoter properties**. *PLoS Genet* 2006, **2**:e54.
11. Ioshikhes IP, Zhang MQ: **Large-scale human promoter mapping using CpG islands**. *Nat Genet* 2000, **26**:61-63.
12. Ku M, Koche RP, Rheinbay E, Mendenhall EM, Endoh M, Mikkelsen TS, Presser A, Nusbaum C, Xie X, Chi AS, Adli M, Kasif S, Ptaszek LM, Cowan CA, Lander ES, Koseki H, Bernstein BE: **Genomewide analysis of PRC1 and PRC2 occupancy identifies two classes of bivalent domains**. *PLoS Genet* 2008, **4**:e1000242.
13. Mikkelsen TS, Ku M, Jaffe DB, Issac B, Lieberman E, Giannoukos G, Alvarez P, Brockman W, Kim TK, Koche RP, Lee W, Mendenhall E, O'Donovan A, Presser A, Russ C, Xie X, Meissner A, Wernig M, Jaenisch R, Nusbaum C, Lander ES, Bernstein BE: **Genome-wide maps of chromatin state in pluripotent and lineage-committed cells**. *Nature* 2007, **448**:553-560.
14. Pauler FM, Sloane MA, Huang R, Regha K, Koerner MV, Tamir I, Sommer A, Aszodi A, Jenuwein T, Barlow DP: **H3K27me3 forms BLOCs over silent genes and intergenic regions and specifies a histone banding pattern on a mouse autosomal chromosome**. *Genome Res* 2009, **19**:221-233.
15. Illingworth RS, Gruenewald-Schneider U, Webb S, Kerr AR, James KD, Turner DJ, Smith C, Harrison DJ, Andrews R, Bird AP: **Orphan CpG islands identify numerous conserved promoters in the mammalian genome**. *PLoS Genet* 2010, **6**.
16. Bock C, Paulsen M, Tierling S, Mikeska T, Lengauer T, Walter J: **CpG island methylation in human lymphocytes is highly correlated with DNA sequence, repeats, and predicted DNA structure**. *PLoS Genet* 2006, **2**:e26.
17. Smallwood SA, Tomizawa S, Krueger F, Ruf N, Carli N, Segonds-Pichon A, Sato S, Hata K, Andrews SR, Kelsey G: **Dynamic CpG island methylation landscape in oocytes and preimplantation embryos**. *Nat Genet* 2011, **43**:811-814.

18. Meissner A, Mikkelsen TS, Gu H, Wernig M, Hanna J, Sivachenko A, Zhang X, Bernstein BE, Nusbaum C, Jaffe DB, Gnirke A, Jaenisch R, Lander ES: **Genome-scale DNA methylation maps of pluripotent and differentiated cells**. *Nature* 2008, **454**:766-770.

19. Mohn F, Weber M, Rebhan M, Roloff TC, Richter J, Stadler MB, Bibel M, Schubeler D: **Lineage-specific polycomb targets and de novo DNA methylation define restriction and potential of neuronal progenitors**. *Mol Cell* 2008, **30**:755-766.

20. Maunakea AK, Nagarajan RP, Bilenky M, Ballinger TJ, D'Souza C, Fouse SD, Johnson BE, Hong C, Nielsen C, Zhao Y, Turecki G, Delaney A, Varhol R, Thiessen N, Shchors K, Heine VM, Rowitch DH, Xing X, Fiore C, Schillebeeckx M, Jones SJ, Haussler D, Marra MA, Hirst M, Wang T, Costello JF: **Conserved role of intragenic DNA methylation in regulating alternative promoters**. *Nature* 2010, **466**:253-257.

21. Das R, Dimitrova N, Xuan Z, Rollins RA, Haghighi F, Edwards JR, Ju J, Bestor TH, Zhang MQ: **Computational prediction of methylation status in human genomic sequences**. *Proc Natl Acad Sci U S A* 2006, **103**:10713-10716.

22. **EpiExplorer supplementary website** [http://epiexplorer.mpi-inf.mpg.de/supplementary/]
